# A singular case of primary aorto-duodenal fistula without pre-existing abdominal aortic aneurism: why and when you should suspect it

**DOI:** 10.1259/bjrcr.20210143

**Published:** 2021-10-20

**Authors:** Giovanna Vacca, Claudia De Berardinis, Salvatore Cappabianca, Angelo Vanzulli

**Affiliations:** 1 Department of University of Campania “Luigi Vanvitelli”, Caserta, Italy; 2 Department of Radiology, University “La Statale” of Milan, Milan, Italy

## Abstract

Although gastrointestinal hemorrhage from aorto-enteric fistulae (AEF) secondary to previous aortic grafts are well known, a primary aorto-enteric fistula (PAEF) without aortic aneurysm is an extremely rare event resulting in poor prognosis and outcome. PAEF is a rare cause of gastro-intestinal (GI) bleeding that radiologists should consider because often its presence is not easily guessed by clinical features. It is difficult to detect at CT examination therefore PAEF might be not diagnosed until a laparotomy. We report a case of a 74-year-old Italian male who presented to our Emergency Department (ED) with brightly red rectal bleeding that occurred from some hours and a pre-syncopal episode. There was no history of analgesic abuse, peptic ulceration, alcohol excess, and weight loss. Standard resuscitation was commenced with the hope that common sources of bleeding such as peptic ulcers or varices would eventually be discovered by endoscopy and treated definitely. An upper GI endoscopy showed brightly red blood in the stomach and in the first portions of duodenum, but no source of active bleeding was found. Diagnosis of PAEF was made by CT and after confirmed during surgical intervention. Both the duodenum and the aorta were successfully repaired by direct suture and synthetic graft replacement, respectively. Diagnosis of primary aortic duodenal fistula (ADF) has been very difficult in this case especially because our patient did not have abdominal aortic aneurism (AAA) history confirmed by CT examination. Radiologist should remember that upper GI bleeding could however be determined by primary ADF also if atherosclerotic damage is severe as in this case. A technically good and complete exam is mandatory to achieve this rare and complex diagnosis. Particularly, an ultra-tardive acquisition phase (5 min after contrast administration) could be helpful to suspect the presence of PADF: the appearance of contrast into the duodenal lumen is an evocative sign useful to increase clinical and radiological suspicious of ADF. Gl bleeding should be assumed to be caused from a PAEF unless another source can be identified without delay. A timely and accurate diagnosis of primary AEF may be challenging due to insidious episodes of GI bleeding, which are frequently under diagnosed until the occurrence of massive hemorrhage.

## Clinical presentation

A 74-year-old male arrived to our ED with brightly red rectal bleeding that occurred from some hours and a pre-syncopal episode.

Twenty days before this event, he visited another hospital with the same clinical presentation and clinicians suggested him to do an upper GI endoscopy privately.

In the last 20 days, he referred some episodes of melena.

The patient has a history of ischemic heart disease in treatment with cardio-aspirin, bladder cancer in follow-up, high blood pressure, dyslipidemia and chronic pain to the lower limb.

On arrival in our ED, vital signs were: a respiratory rate of 22 breaths/min, a heart rate of 80 beats/min, a blood pressure of 95/60 mm Hg, and a body temperature of 36.3°C.

Physical examination revealed a massive brightly red rectal bleeding, no abdominal pain, pale conjunctiva, no heart murmur, and clear breathing sound. The abdomen was soft and there was no visceromegaly.

His initial hemoglobin value was 7.3 g dl^−1^, glycemia 396 mg dl^−1^, lactate 16 mg dl^−1^, pH 7.12, HCO3^-^ 10, and BE^-^ 17.

He was resuscitated with fluid and 4 units of packed red blood cells were administered. A nasogastric tube was positioned with the discovery of bright red blood.

While the patient was about to be taken for an emergent endoscopy, clinical signs of hemorrhagic shock have become more serious: patient had a very low arterial blood pressure that was no more measurable, his heart rate amounted to 120 beats/min. Patient presented cold and marbled skin.

The patient was intubated in the emergency department and noradrenaline (0.2 mcg/kg/min) was administered.

## Investigations

Standard resuscitation was predisposed waiting for upper GI endoscopy with the hope that common sources, either peptic ulcers or varices would eventually be founded and stop bleeding.

Upper GI endoscopy showed the esophagus with traces of bright red blood and stomach full of clots and food debris, without definite sources of active bleeding. A continue supply of blood and bright red clots was observed from the distal duodenum but the device was not able to overstep the second portion of the duodenum.

For this reason, CT examination with contrast medium was necessary to identify the cause of GI bleeding.

Computed tomographic angiography represents a diagnostic tool for ADF.^1^

**Figure 1.**
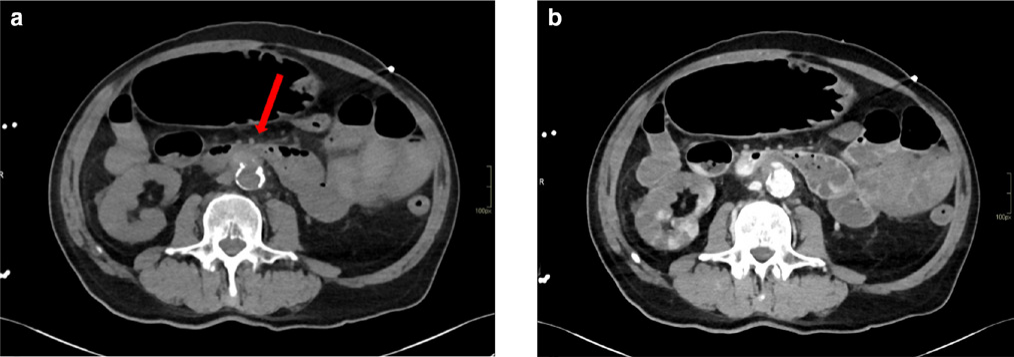
A 74-year-old Italian male who presented to our Emergency Department (ED) with bright red rectal bleeding that occurred from some hours and a pre-syncopal episode a) The no-contrast phase showed widespread spontaneous hyper-density of the bowel lumen content especially at the jejunum-ileum. (**b**) In the arterial phase, abdominal aorta presented regular diameters and course but diffuse and severe signs of parietal atheromasia with multiple calcific and ulcerated plaques especially in thearterial phase, abdominal aorta presented regular diameters and course but diffuse and severe signs of parietal atheromasia with multiple calcific and ulcerated plaques especially in the sub-renal tract: at this level, the aortic wall was thickened on the antero-lateral right side (12 mm). Multiples shaded areas of hypo-density of the left kidney as marker of hemorragic shock.

**Figure 2.**
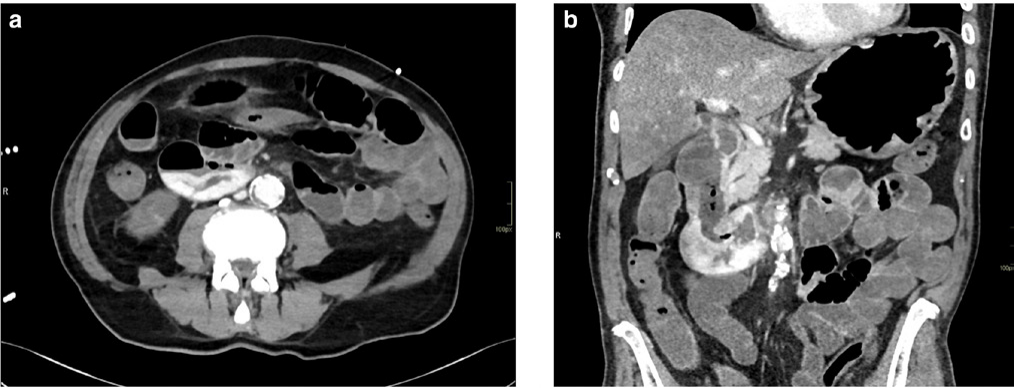
CT late phase of the study (respectively in axial and coronal plane) shows an active spreading of contrast medium in the third-fourth portion of duodenum.

After the upper GI endoscopy, abdomen CT was performed using a classic quadri-phasic protocol of study. Particularly, this protocol consisted of a no-contrast phase (Figure 1a) followed by arterial phase, venous phase, and later phase acquired respectively 35 s (Figure 1b), 90 s, 180 s after contrast-medium administration. For this specific clinical issue, in order to highlight the possible presence of GI bleeding, another ultra-late phase (5 min after contrast-medium administration) was executed: it was particularly helpful to detect the active bleeding (Figure 2).

The no-contrast phase showed widespread spontaneous hyperdensity of the bowel lumen content especially at the jejunum-ileum. (Figure 1a).

Stomach and duodenum were overextended by air because of the recent endoscopic exam.

There was no evidence of free intra-peritoneal air or fluid.

Abdominal aorta presented regular diameters and course but diffuse and severe signs of parietal atheromasia with multiple calcific and ulcerated plaques especially in the sub-renal tract: at this level, the aortic wall was thickened on the antero-lateral right side (12 mm) with hyper-density on the no-contrast phase and minimal inhomogeneity of the periaortic fat (Figure 1b).

This portion of the abdominal aorta was localized in proximity to the third portion of duodenum without any sign of aortic wall interruption, free gas on the periaortic fat or a conduit between duodenum and aorta.

The right iliac common artery was occluded.

In the late and ultra-late phase of the study, an active spreading of contrast medium was evident in the third-fourth portion of duodenum (Figure 2).

Signs of hemorrhagic shock were represented by multiple-shaded areas of hypo-density and hypo-perfusion of liver, adrenal glands, spleen, and kidneys (Figure 1b). The inferior vena cava and the portal vein appeared filiform.

Patient was then taken to the interventional radiology suite where selective angiography of the superior mesenteric artery and duodenal branches was performed without showing any active bleeding.

He was taken to the operating room for an emergent laparotomy.

## Differential diagnosis

The most common cause of upper GI bleeding is represented by peptic ulcer disease and accounts for up to 67% of upper GI bleeding cases.^2^ When a GI bleeding is suspected, clinical and anamnestic information should be searched regarding the nature of bleeding (*e.g.* haematemesis), bleeding diathesis (*e.g.* von Willebrand disease), comorbidities and systemic symptoms (*e.g.* vasculitis, amyloidosis), drug use (*e.g.* non-steroidal anti-inflammatory drugs), prior procedures (*e.g.* abdominal aortic aneurism repair) and prior radiation and family history (*e.g.,* malignancies).^2^ This research could be effective to identify the underlying etiology^3^

Endoscopy is often the initial exam for any suspected upper GI bleeding because it confirms the source of bleeding and tries eventually to stop the bleeding.^2^

In our case, a huge quantity of blood and a large bright red clot’s burden filled the stomach and duodenum and the endoscopic tube did not go beyond the second portion of the duodenum: the exact source of bleeding was not identified on endoscopy.

CT examination was then performed after upper GI endoscopy. Considering also the anamnestic information collected by clinicians, other causes of gastro-intestinal bleeding could have been represented for exclusion by third-forth portion duodenal ulcer and perforation, a gastro-intestinal tumor, primary aorto-enteric fistula.

## Treatment

In the operating room, a midline laparotomy incision was made. Any fluid or blood was found into the peritoneal cavity. Stomach, duodenum, and ileum were tensely distended. A longitudinal gastrotomy along the anterior portion of the stomach was made. Copious amounts of fresh blood and old clots were encountered. At the level of the fourth portion of duodenum, a hard adhesion was evident between anterior aortic wall and posterior duodenum wall; the duodenum lumen presented a focal weakness of 1 cm that was related to a possible PAEF. The breach was reconstructed with a dacron silver prothesis although proximal and distal anastomosis. Prothesis was then covered by retroperitoneal tissue. Later the third and fourth portions of duodenum were resected.

## Outcome and follow-up

Unfortunately, the patient expired few hours after surgical intervention. This event was due to patient’s critical conditions on his admission to our hospital before surgical intervention. We have to keep in mind that AEPF is a life-treating condition. Insidious episodes of GI bleeding, as in this case, are frequently underestimated leading to the sudden occurrence of massive hemorrhage.

## Discussion

Aorto-enteric fistulae (AEF) consists on a direct and pathologic communication between the lumen of aorta and any portion of the gastrointestinal tract.^3^

The duodenum, especially in the third and fourth portion, for his anatomic position and the greater contact surface with the abdominal aorta, is the most common site of fistulas representing the 80% of the AEF.^3^ Less common sites include proximal small bowel, colon, and esophagus.^2^

Generally, AEF can be divided into primary (PAEF) and secondary types (SAEF).

PAEF occur in patients with no previous aortic surgery or trauma. There are some pathologic conditions that predispose PAEF like atherosclerotic abdominal aortic aneurysms (AAA) or penetrating atherosclerotic ulcers. Atherosclerotic changes are found in 85% of AEF-related AA.^4^ Less common causes can be inflammatory or infectious aortitis, diverticulitis, appendicitis, radiations, foreign bodies, and gastrointestinal tumors.^2^

SAEF usually results from a previous aortic aneurysm grafting and are caused by a direct erosion of the aortic prosthesis into the GI tract.^3^

SAEF are 10 times more frequent than PAEF^1^; autopsy reports suggest an incidence of 0.02–0.07% of primary fistulas and 1% in patients with abdominal aorta reconstructions.^5^

PAEF is a rare and life-threatening condition due to the non-specific clinical presentation, causing a mortality ranging from 33 to 85%.^4^ The most important prognostic factor is by far PAEF’s early diagnosis.^6^ PAEFs are clinically revealed by GI bleeding in approximately 80% of the cases but the classic triad of GI bleeding, pulsating abdominal mass and abdominal pain occurs only in 25% of PAEF cases.^4^

Other rare symptoms include intermittent back pain, fever, sepsis, melena, and syncope.^7^

The first GI bleeding known as “herald bleeding” is usually self-limited, due probably to a transient bleeding from a small fistula stopped by the formation of a blood clot, and it is followed by a secondary massive bleeding, when the clot is removed, occurring within the next six hours in one-third of cases.^8^

In our case, the patient had a “Herald” bleeding 20 days prior to the catastrophic hemorrhage, when bright red rectal bleeding occurred for the first time.

Considering that the onset of symptoms could be even more challenging with anemia as the only indicator of GI bleeding, clinical suspicion is very important in the diagnosis of AEF.

In a normotensive patient, upper gastrointestinal endoscopy should be the initial step in the search for a cause.

However, lesions without active bleeding founded through endoscopy do not rule out an ADF^9,10^; moreover, as in our patient, the length of the endoscope does not permit the visualization of the distal duodenum where ADF often occurs.^6^

CT with intra-venous contrast is probably the most useful diagnostic tool to diagnose PAEF; the most common signs that strongly suggest a PAEF are the loss of continuity and air bubbles in the aneurysm wall that are pathognomonic^11^ or destruction of the fat plane between the aneurysm and duodenum and the visualization of the contrast within the GI lumen.^7^

Regarding this patient, we have no clinical suspicious neither of secondary AEF because patient have not undergone to previous surgical intervention or primary AEF because he did not have AAA history confirmed by CT examination. We would like to underline that CT diagnosis of primary ADF could be even more difficult. We should keep in mind that upper GI bleeding could however be caused by ADF if atherosclerotic damage is severe as in this case.

An ultra-tardive acquisition phase (5 min after contrast administration) could be helpful to imagine the presence of PADF: the appearance of contrast into the duodenal lumen is an evocative sign useful to increase clinical and radiological suspicious of ADF. Any aneurysm, loss of continuity, and air bubbles were founded.

In case of unknown GI bleeding, we suggest to perform a complete angiography rather than a selective angiography because, as in this case, ADF could be not seen; oblique projections might be helpful for this purpose.

Despite technological advances in endoscopy and imaging, a prompt diagnosis should be guided by a clinical suspicion; surgical repair without delay is the only possibility for survival.^5^

## Learning points

- PADF is by definition caused by direct and pathologic communication between the lumen of aorta and any portion of the gastrointestinal tract. Atherosclerotic change is found in 85% of AEF-related AAA.^12^
- Even if CT sensitivity is very low for diagnosis ADF, we should think of its presence especially if other main causes of upper gastrointestinal bleeding are excluded
- Performing an ultra-tardive acquisition phase (5 min after contrast administration) is helpful to demonstrate PADF because appearance of contrast in the duodenal lumen is an evocative sign to increase clinical and radiological suspicious of ADF.
- When possible, CT is useful before surgical exploration to examine the third and forth portion of duodenum especially when EGD is unconclusive.
